# Injury of photoreceptors and retinal pigment epithelium in macular area of a preterm infant: A case analysis

**DOI:** 10.1097/MD.0000000000021096

**Published:** 2020-07-17

**Authors:** Xiantao Sun, Ting Liu, Shuang Sun, Yuebing Lu, Fei Wang, Yuanchun Xie, Guanfeng Li, Hui Wang, Yu Jiang, Yunyun Huang

**Affiliations:** Department of Ophthalmology, Henan Children's Hospital, Children's Hospital Affiliated of Zhengzhou University, Zhengzhou, China.

**Keywords:** photoreceptors injury, preterm infant, retinal pigment epithelium injury, transmitted fluorescence

## Abstract

**Rational:**

Injury of photoreceptors and retinal pigment epithelium (RPE) in macular area of premature infants is very rare.

**Patient concerns:**

A preterm infant delivered under general anesthesia. The infant was born at 28 weeks’ and 4 days’ gestation, with a birth weight of 1.15 kg and a treatment of oxygen inhalation after birth. According to the related protocol formulated by the Ophthalmology Branch of the Chinese Medical Association in 2014, the infant was regularly checked in our hospital.

**Diagnosis:**

Optical coherence tomography (OCT) examination showed injuries of the photoreceptors and RPE in macular area.

**Interventions:**

The fundus screening at 40 weeks’ and 4 days’ gestation (corrected gestational age) showed retinopathy of prematurity in bilateral eyes, with round yellow-white lesions at the macular area of right eye and sub-temporal macular area. OCT examination showed interrupted signals in the external limiting membrane (ELM), inner segment of the photoreceptors (IS)/outer segment of the photoreceptors (OS) layer, interdigitation zone (IZ), and RPE of the central fovea of macula of the right eye, with the area of defect of approximately 184 μm. Enhanced signal reflection was found under the defect area. Interrupted signals were also found in the IS/OS layer of the central fovea of macula of the left eye, with the area of defect of approximately 222 μm. Fundus fluorescence angiography (FFA) examination showed transmitted fluorescence at the macular area of the right eye and sub-temporal macular area of the left eye, suggesting retinopathy of prematurity in bilateral eyes.

**Outcomes:**

Several factors, such as photic damage, eye injuries, hyperpyrexia, and underlying diseases, could cause macular retinal injuries. However, the baby had not received any radiation from high energy intense light sources, and had no history of hyperpyrexia or trauma. Fundamental screening was performed 1 year and 4 months of age and no obvious change was found in the round yellow-white lesions of the eyes compared with that in earlier stages. We have contacted with the patient for the follow-up OCT and FFA examinations a month later to check the possible structural changes of the macular area.

**Lessons:**

The retina of a preterm infant is underdeveloped, we speculated that the bilateral retinal injuries in this baby could be caused by various factors.

## 1 Introduction

Injury of the photoreceptors and retinal pigment epithelium (RPE) in macular area is very rare. Previous studies have reported that this disease is associated with photic damages due to an evident exposure history, and the damages from arc light and laser are the most serious and common.^[1,2]^ To the best of our knowledge, no reports on neonatal retinal injuries have been published. Herein, we reported a preterm infant delivered under general anesthesia, whose optical coherence tomography (OCT) examination showed injuries of the photoreceptors and RPE in macular area. These findings have important significance for studies on retinal injuries in preterm infants and retinal development.

## 2 Case presentation

This study was approved by the Ethics Committee of the Henan Children's Hospital, and informed consent was obtained from the parents of the baby. The baby was a singleton, and the second birth from the third pregnancy. The ethnicity of the baby was Han. The baby was spontaneously delivered pre-term at the gestational age of 28 weeks and 4 days, on August 25, 2018, due to premature rupture of membrane for 36 hours and oligohydramnios. The body weight of the baby at birth was 1150 g. The baby started crying at 2 minutes after delivery, with no history of asphyxia or meconium contamination of the skin. The 1, 5, and 10 minutes-Apgar scores of the baby were 7, 8, and 8, respectively. The baby was immediately admitted after delivery to the Preterm Neonatal Department of the Henan Children's Hospital for dyspnea for 4 hours. The systemic physical examinations showed no deformation. The mother of the baby was healthy, with no history of any drug use during the pregnancy. The mother reported no consanguineous marriage, or histories of hereditary, and congenital diseases. The baby received blue-light radiation 6 times every 2 to 3 days for neonatal hyperbilirubinemia. Five times, the radiation lasted for 12 hours, while the sixth time, the radiation lasted for 24 hours. The bilateral eyes of the baby were covered by a black eyeshade during the radiation therapy.

**Figure 1:**
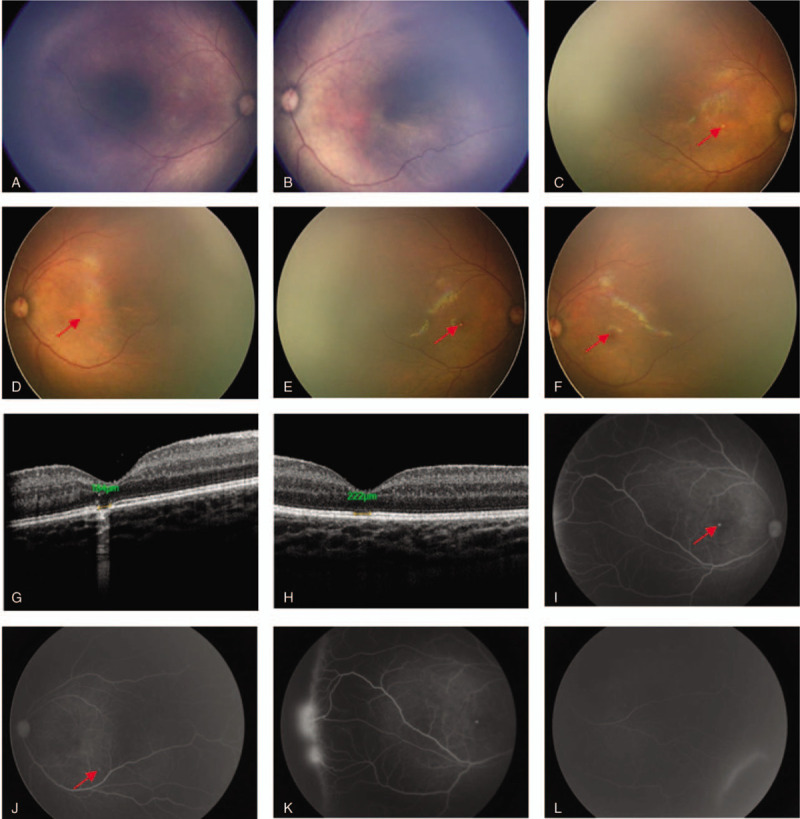
A: Fundus photography of the right eye after the first pupil dilation (corrected gestational age of 32 weeks and 2 days). B: Fundus photography of the left eye after the first pupil dilation (corrected gestational age of 32 weeks and 2 days). C: Fundus photography of the right eye after pupil dilation (corrected gestational age of 40 weeks and 4 days). D: Fundus photography of the left eye after pupil dilation (corrected gestational age of 40 weeks and 4 days). E: Fundus photography of the right eye after pupil dilation (corrected gestational age of 45 weeks and 2 days). F: Fundus photography of the right eye after pupil dilation (corrected gestational age of 45 weeks and 2 days). G: OCT image of the macular area of right eye. H: OCT image of the macular area of left eye. I and K: FFA images in the venous phase of the right eye. J and L: FFA images in the venous phase of the left eye. Arrows show the lesions. FFA = fundus fluorescence angiography, OCT = optical coherence tomography.

Fundus screening for preterm birth is routinely conducted for the baby at our hospital. The first examination was conducted at the corrected gestational age of 32 weeks and 2 days. After compound tropicamide eye drops were administered for pupil dilation, PanoCam fundus screening showed no abnormality at the anterior segment of bilateral eyes. The boundaries of the optical disks at bilateral fundus were clear, and the sizes and color were normal. The flow of the blood vessels in the I and II areas of retina were normal. The retinal blood vessel in both eyes reached the II area, while in the III area, the blood vessels did not reach the ora serrata (Fig. 1A and B). Re-examination was conducted every 2 to 3 weeks until the corrected gestational age was 40 weeks and 4 days. The RetCam fundus screening showed no abnormalities at the anterior segment of bilateral eyes. The boundaries of the optical disks at bilateral fundus were clear, and the sizes and color were normal. The flow of the blood vessels in the I and II areas of retina were normal. In the II to III areas at the temporal side of bilateral eyes, 3 to 4 separation lines were found. White punctate lesions were found in the macular area of the right and sub-temporal macular area of the left eye, and the local pigments were uneven (Fig. 1C and D). The baby was re-examined every 2 to 3 weeks. The RetCam fundus screening after 1 month showed no abnormalities in the I area of bilateral eyes. The macular structure was normal, blood vessels in the II area were circuitous, while 3 to 4 ridges were found in the temporal side close to the III area of the right eye, with exudation anterior to the rigids, and a non-blood vessel area posterior to the rigids. Two separation lines were found in the super-temporal area of the left eye, with some blood vessels passing through the separation lines. The pigments in the macular area were uneven in the right eye, and a macular hole was suspected in this area. Then, OCT and fundus fluorescein angiography were conducted under general anesthesia (Fig. 1E and F). OCT showed interrupted signals in the external limiting membrane (ELM), IS/OS layer, interdigitation zone (IZ), and RPE of the central fovea of macula of the right eye, with the area of defect of approximately 184 μm. Enhanced signal reflection was found under the defect area (Fig. 1G). Interrupted signals were also found in the IS/OS layer of the central fovea of macula of the left eye, with the area of defect of approximately 222 μm. FFA examination showed transmitted fluorescence at the macular area of the right eye and sub-temporal macular area of the left eye. The intensity of fluorescence gradually increased with time, while the shapes remained unchanged. Increased number of peripheral capillaries were observed in the right retina, which was circuitous with uneven thickness, and showed a broom-shaped distribution. The peripheral blood vessels were linked to each other and formed a circuit. Hyperplasia of the fibrous tissues was found at the boundaries of the lesions, which showed ridge-shaped changes with fluorescein staining. Neovascular fibroplasia membrane was found anterior to the ridge, which was accompanied by large amount of transmitted fluorescence, while the boundary between the ridge and distal non-blood vessel area was clear (Fig. 1I and K). The number of peripheral capillaries in the left eye was increased, which showed the shape of parallel brush. Increased permeability of the capillaries was found, and a small amount of transmitted fluorescence was found at late stage. The boundary between the lesion and distal non-blood vessel area was clear (Fig. 1J and L). These findings suggested the following: injuries of photoreceptor layer in the macular area of bilateral eyes; and retinopathy of prematurity in bilateral eyes. Electrophysiological examination of the visual evoked potential (VEP) showed delayed latency of P_2_ waves in bilateral eyes, while ERG showed decreased amplitudes of all the reactions. After vitamin B, lutein, and vitamin AE treatments were administered, OCT after 2 months showed no significant changes in the lesions at the central fovea of macula.

## 3 Discussion

According to the “Guidelines for the Screening Examination of Premature Infants for Retinopathy of Prematurity in China” published in 2004 and 2014, our hospital will conduct fundus screening for preterm and low-birthweight infants with a birth weight of <2000 g or a gestational age of <32 weeks and follow up to vascularization of the peripheral retina.^[3,4]^ Since 2009, we started using indirect ophthalmoscopy to perform fundus screening for preterm infants who meet the guidelines. Since 2011, we began to use Retcam3 with indirect ophthalmoscopy for fundus screening of preterm infants. In 2012, our hospital was approved as “Zhengzhou Premature Retinopathy Screening Center.” In 2015, the use of Panocam (Vianas Medical Devices Co., Ltd., PNLT-10-10-22) was increased, and we also purchased the children's fundus angiography system (Retcam3 system configuration), horizontal OCT (Clinico Medical Instrument Technology Co., Ltd, iVue100). Under general anesthesia, fundus angiography is performed for children who have difficulties to cooperate with the examination.

Previous studies indicated that several factors could cause injuries of the macular photoreceptor layer as follows: photic damage, including damages caused by sunlight, arc light from welding, laser, ultraviolet radiation in plateau areas, LED light, and operation microscope^[5–10]^; history of ocular disease or systemic diseases, including high myopia, post-central serous chorioretinopathy, glaucoma, and diabetes^[11]^; cranial or ocular trauma^[12,13]^; and hyperpyrexia.^[14]^ Although we considered that congenital hypoplasia could induce such a disorder, there are no reports supporting this hypothesis. In addition, we had never found similar cases in the medical records of the patients who received fundus screening in >10 years at our hospital.

The baby in this report had lesions in bilateral eyes. OCT showed that the lesions were at the ELM, IS/OS layer, IZ, and RPE of the central fovea of macula of the right eye. In addition, IS/OS layer disruption was found in the left eye. FFA showed transmitted fluorescence at the macular area of the right eye and sub-temporal macular area of the left eye. The locations of the lesions shown in OCT and FFA in the right eye were identical; however, for the left eye, FFA showed that the lesion was at the sub-temporal macular area, while OCT scanning was relatively difficult after general anesthesia. Thus, we suspected that the transmitted fluorescence at the sub-temporal macular area of the left eye could be the RPE injuries. After reviewing the images of the first fundus photography that showed no sign of round yellow-white lesion; however, uneven pigments at the macular area were found in bilateral eyes. In addition, the pigments at the macular area were more uneven after 2 months.

The baby's family reported no history of irradiation exposure during the pregnancy, as well as no histories of hyperpyrexia and trauma after delivery. The baby was spontaneously delivered, and the birth process was smooth, with no history of birth injury or use of obstetric forceps. However, the baby received radiation from blue-light, Retcam, and Panocam. Both eyes of the baby were covered by black eyeshade during the blue-light radiation. For the Retcam, halogen light source with constant brightness was used, and the emission spectrum of the light was 300 to 1100 nm. While for the Panocam, LED solid state light source was used, and the emission spectrum of the light was 400 to 800 nm. Retinal injuries caused by Retcam or Panocam have not been previously reported. However, the blue-light from the LED lights, cell phones, LED screens of computers, which are very commonly used in daily lives, could increase the free radicals in the retina, and thus induce retinal oxidative stress and oxidative damages. Lin et al^[15]^ investigated the photic damage caused by the radiation from laser pointer, and found that the disruptions of MZ, ellipsoid zone (EZ), and IZ were irreversible, and these tissues lacked the capability to regenerate, which were in agreement with our findings.

Tran-Viet et al^[16]^ used handheld OCT to scan the macula of babies with ROP or hypoxic-ischemic encephalopathy (HIE), and found that such babies (with the corrected gestational ages of 35–42 weeks) had cystoid macular edema and underdeveloped macular photoreceptor layer, and the range of lesions was relatively large. In contrast, the lesion of the baby in this report was restricted at the local area; however, several different tissues were involved, such as the absence of photoreceptor layer, and lesions of the ELM, IZ, and RPE.

Macular hole refers to the complete or partial absence of the tissues from the retinal inner limiting membrane to photoreceptor layer, and the ocular fundus shows an oval hole with sharp margin. The size of the hole varies in different cases. Macular hole is mainly idiopathic, which is associated with the traction of macula by vitreous body, or with trauma in some rare cases. Macular hole is occasionally accompanied by the absence of IS/OS layer of the central fovea of macula, and the patients could not achieve complete recovery after the hole is sealed by operation.^[10,17,18]^

Diseases of retinal degeneration or hereditary metabolic retinal malnutrition, such as abetalipoproteinemia, could induce injuries of photoreceptors and pigment epithelium.^[19]^ For instance, in the early stage of vitelliform macular dystrophy (Best disease), the macular area is relatively normal, or with mottling pigment disorder and abnormal EOG, while OCT could identify restricted local discontinuity of the IS/OS layer and high-reflection non-cellular dense substances. While in the late stage, vitelline-like deposition could be found in the macular area, which could degrade to an appearance of fried egg.^[20,21]^ Cone dystrophy is a hereditary retinal degeneration with the involvement of the functions of cone cells. The fundus screening could show round or bull's eye-shaped degradation in the macular area, while FFA could show transmitted fluorescence or bull's eye-shaped hyper-fluorescence in the macular area. The characterized manifestations include near disappearance of ERG of cone cell response, while ERG of light adaptation is normal.^[11,22]^ The baby in this report had no traction of macula by vitreous body, underlying diseases, or history of trauma, while the lesion was restricted, with no macular hole. Interlayer scanning of the retina did not show high-reflection dense substances, and no vitelline-like deposition was found in the late stage. The amplitudes of all the ERG responses decreased but did not disappear, which were inconsistent with the pathogenesis of Best disease or cone dystrophy.

Previous studies have reported that 48.0% of the cases with no macular IS/OS layer are due to unknown causes.^[7]^ Therefore, we could not rule out the possibility of retinal dysplasia in this baby. Several factors are reported to induce macular photoreceptor layer injuries. However, the baby in this report had no evident causes of photoreceptor and pigment epithelium injuries. In addition, the characteristics of the baby were also different from previous cases. For instance, a preterm infant has underdeveloped functions of the organs and systems, along with vitamin deficiencies, especially vitamin A, which is responsible for maintaining normal visual functions, and vitamin E, which plays an important role in anti-oxidation. A preterm infant is more susceptible to external physical and chemical factors. Thus, the retinopathy in bilateral eyes of the baby could be caused by various factors or retinal dysplasia. The patient in this case report was a preterm infant, and no similar cases have been reported to date. Therefore, this is the first report of retinal injury in a preterm infant, which could have profound significance in preventing retinal injuries in preterm infants, as well as in the studies on retinal development. As the age of this infant is extremely young, no follow-up data are available yet. We will continue to contact the patient for further follow-up.

## Author contributions

**Conceptualization:** Xiantao Sun.

**Data curation:** Shuang Sun, Ting Liu.

**Formal analysis:** Shuang Sun, Ting Liu, Yuebing Lu.

**Investigation:** Fei Wang, Yuanchun Xie, Guanfeng Li, Hui Wang, Qiuli Zhang, Honghui Cao.

**Methodology:** Xiantao Sun.

**Resources:** Xiantao Sun, Ting Liu.

**Validation:** Xiantao Sun, Hui Wang.

**Visualization:** Yu Jiang, Yunyun Huang.

**Writing–original draft:** Xiantao Sun, Ting Liu, Shuang Sun.

**Writing–review & editing:** Xiantao Sun.

## References

[1] Organisciak DT Vaughan DK Retinal light damage: mechanisms and protection. Prog Retin Eye Res 2010;29:113–34. 19951742 10.1016/j.preteyeres.2009.11.004 PMC2831109

[2] Wang MY Li YR Wang GL Morphological changes of photic maculopathy in fourier-domain optical coherence tomography. Zhong Hua Yan Wai Shang Zhi Ye Yan Bing Za Zhi 2014;36:340–3.

[3] Ocular Fundus Diseases Group, Chinese Ophthalmologic Society, Chinese Medical Association. Guidelines for the screening examination of premature infants for retinopathy of prematurity in China. Zhong Hua Yan Ke Za Zhi 2014;50:933–5.

[4] Li XX Characteristics and screening guideline of retinopathy of prematurity patients in China. Zhong Hua Yan Di Bing Za Zhi 2004;20:384–6.

[5] Shahinfar S Edward DP Tso MO A pathologic study of photoreceptor cell death in retinal photic injury. Curr Eye Res 1991;10:47–59. 10.3109/02713689109007610 2029848

[6] Lee GD Baumal CR Lally D Retinal injury after inadvertent handheld laser exposure. Retina 2014;34:2388–96. 25380069 10.1097/IAE.0000000000000397

[7] Lo Giudice G Catania AG Galan A Adaptive optics study of photoreceptors layer damage from presumed sun exposure: a case report. Indian J Ophthalmol 2016;64:860–1. 27958216 10.4103/0301-4738.195619 PMC5200995

[8] Henry MM Henry LM Henry LM A possible cause of chronic cystic maculopathy. Ann Ophthalmol 1977;9:455–7. 869422

[9] Yang LY Han M Xu YS Preliminary analysis of the photoreceptor inner/outer segment (IS/OS) layer local deletion in macular. Zhong Guo Shi Yong Yan Ke Za Zhi 2013;31:267–9.

[10] Comander J Gardiner M Loewenstein J High-resolution optical coherence tomography findings in solar maculopathy and the differential diagnosis of outer retinal holes. Am J Ophthalmol 2011;152:413.e6–9.e6. 21708377 10.1016/j.ajo.2011.02.012

[11] Zhang CF Zhang FT Chen YX Disease of Ocular Fundus. Beijing: People's Medical Publishing House; 2017.

[12] Wang GL WWB. Traumatic ocular fundus disease. Optical Coherence Tomography: Atlas for the diagnosis of Ocular fundus diseases. 2009;Beijing: Beijing Science and Technology Press, 264–265.

[13] Hu YT Li Q Ma XQ Retinal photoreceptor layer disruption caused by ocular blunt trauma: a case report. Zhong Hua Yan Di Bing Za Zhi 2013;29:93–193.

[14] Lu Y Zhao JL Two cases of IS/OS layer of macular photoreceptors after hyperpyrexia. Zhong Hua Yan Di Bing Za Zhi 2011;27:395–6.

[15] Lin B Huang Y Liu XL Optical coherence tomography characteristics of macular injury from laser spot irradiation. Zhong Hua Yan Shi Guang Xue Yu Shi Jue Ke Xue Za Zhi 2016;18:627–30.

[16] Tran-Viet D Wong BM Mangalesh S Handheld spectral domain optical coherence tomography imaging through the undilated pupil in infants born preterm or with hypoxic injury or hydrocephalus. Retina 2018;38:1588–94. 28570486 10.1097/IAE.0000000000001735 PMC5708150

[17] Inoue M Watanabe Y Arakawa A Spectral-domain optical coherence tomography images of inner/outer segment junctions and macular hole surgery outcomes. Graefes Arch Clin Exp Ophthalmol 2009;247:325–30. 19018552 10.1007/s00417-008-0999-9

[18] Chung H Byeon SH New insights into the pathoanatomy of macular holes based on features of optical coherence tomography. Surv Ophthalmol 2017;62:506–21. 28300548 10.1016/j.survophthal.2017.03.003

[19] Verbakel SK van Huet RAC Boon CJF Non-syndromic retinitis pigmentosa. Prog Retin Eye Res 2018;66:157–86. 29597005 10.1016/j.preteyeres.2018.03.005

[20] Querques G Regenbogen M Quijano C High-definition optical coherence tomography features in vitelliform macular dystrophy. Am J Ophthalmol 2008;146:501–7. 18619572 10.1016/j.ajo.2008.05.029

[21] Guziewicz KE Sinha D Gomez NM Bestrophinopathy: an RPE-photoreceptor interface disease. Prog Retin Eye Res 2017;58:70–88. 28111324 10.1016/j.preteyeres.2017.01.005 PMC5441932

[22] Sahel JA Marazova K Audo I Clinical characteristics and current therapies for inherited retinal degenerations. Cold Spring Harb Perspect Med 2014;5:a017111. 25324231 10.1101/cshperspect.a017111 PMC4315917

